# An overview of factors affecting the quality of beef meatballs: Processing and preservation

**DOI:** 10.1002/fsn3.2812

**Published:** 2022-04-05

**Authors:** Xiangren Meng, Danxuan Wu, Zhaoli Zhang, Hengpeng Wang, Peng Wu, Zhicheng Xu, Ziwu Gao, Benjamin Kumah Mintah, Mokhtar Dabbour

**Affiliations:** ^1^ 38043 College of Food Science and Engineering Yangzhou University Yangzhou China; ^2^ CSIR ‐ Food Research Institute Accra Ghana; ^3^ Department of Agricultural and Biosystems Engineering Faculty of Agriculture Benha University Moshtohor Egypt

**Keywords:** beef meatballs, processing, quality, storage and preservation

## Abstract

Beef meatball (BM) is a traditional delicious snack with rich nutrition and unique flavor, making it a preferred choice for most consumers. However, the quality of BM is easily affected by many factors, such as the processing, storage, and preservation, which limit the competitive positioning with respect to its market. Therefore, it is essential to pay attention to each step during the processing of BMs. Based on previous studies, this systematic review focuses on the effect of key processing factors (including raw materials and ingredients, beating, cooking methods, storage, and preservation) on the quality of BMs. Additionally, this study assessed the effect of each process factor on the physicochemical, sensory, nutritional, and safety attributes of BMs. Finally, the existing review will be beneficial in examining/describing the factors impacting the quality of BMs during processing, which would provide theoretical reference and scientific basis for the standardization and industrialization of BMs.

## Graphical abstract

Based on previous studies, this systematic review focuses on the effect of key processing factors (including raw materials and ingredients, beating, cooking methods, storage, and preservation) on the quality of beef meatballs (BMs).

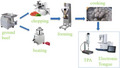

## 1 INTRODUCTION

**FIGURE. 1:**
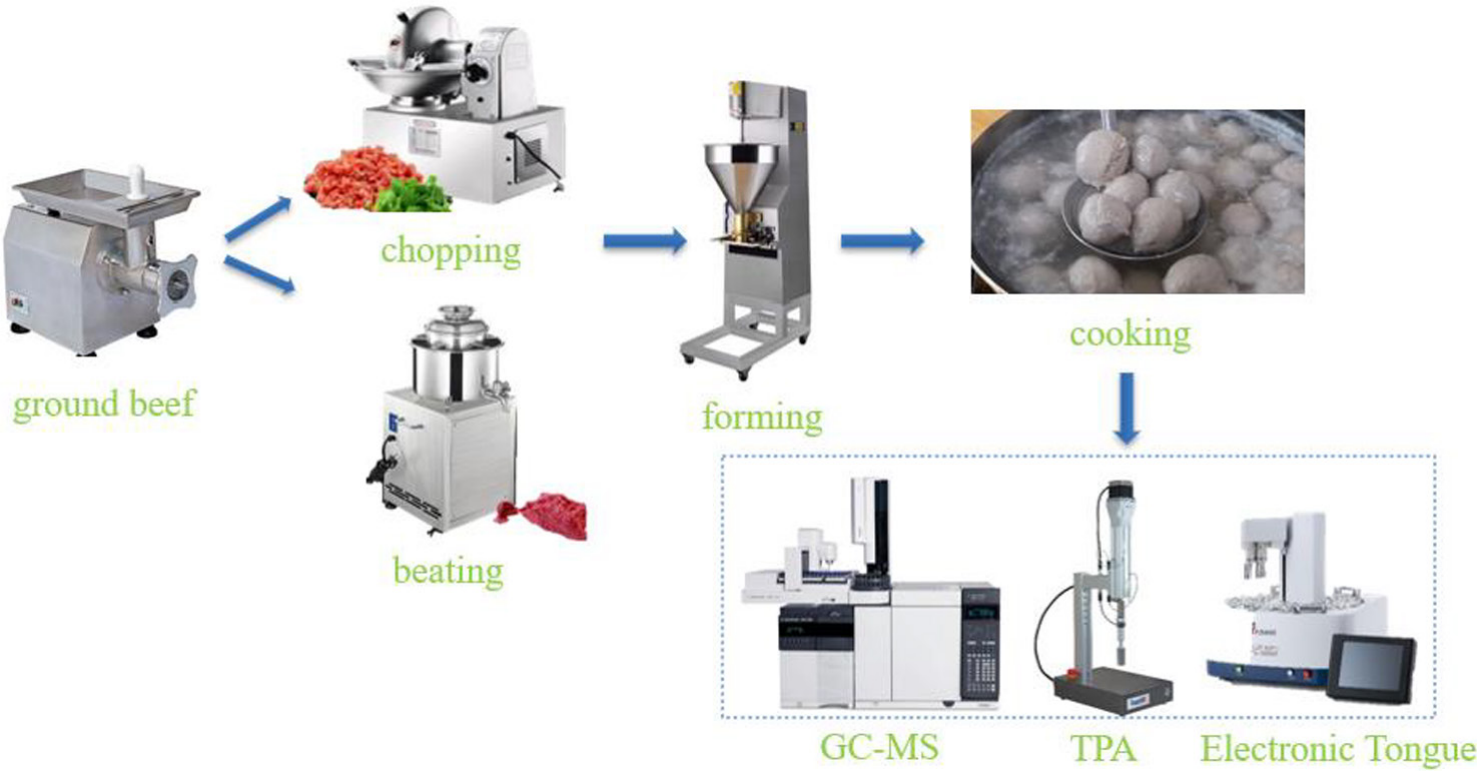
Schematic diagram of the review

Beef is characterized by a high protein content of 22%, while the fat content is only about 10%. The ratio of n‐6/n‐3 polyunsaturated fatty acids (PUFAs) is appropriate, and it also contains a variety of minerals (e.g., phosphorus, potassium kalium, and sodium) and vitamins (e.g., vitamin E, thiamin, and riboflavin). The quality of beef products is related to many factors, such as distinct parts of beef, ingredients, and processing conditions. Beef meatball (BM), an important ready‐to‐eat meat product, is also one of the most common among hot pot materials in China (Cisowska et al., 2014; Rohman et al., 2011). BM is in round form which is prepared by adding water, flour, spices, and fat with minced beef and processed by precooking, storage, and reheating to make suitable for taking. Most consumers have a preference for BMs due to their lower price, more convenient processing, unique taste, and better sensory properties (including cohesiveness, chewiness, and springiness). However, in the processing of BMs, there are some problems like: (1) the production of BMs mostly relies on the experience of technicians and chefs, which leads to a lack of theoretical and/or scientific support for the quality of BMs; (2) the quality of BMs produced by different manufacturers is uneven. The variations in the quality of BMs have led to differences in the stability of minced meat, yield, cooking loss rate, storage period, and sensory quality (texture, juiciness, and flavor of BMs); and (3) the standard processing procedure for BMs had not been established, restricting the industrialization and marketability of BMs (Zhang, Zhang, et al., 2020; Zhang, Zhao, et al., 2020). Presently, few reports on the factors influencing the quality in the processing of BMs exist. In this review, therefore, the research progress in the factors affecting the quality of BMs in three areas, including ingredients, processing process, storage, and preservation, is discussed. Figure 1 shows the schematic diagram of the review. The purpose is to provide theoretical and technological guidance for the production of high‐quality BMs, and the implementation of reasonable quality control procedures for BM production.

## 2 PROCESSING PROCEDURES OF BEEF MEATBALLS

**FIGURE. 2:**
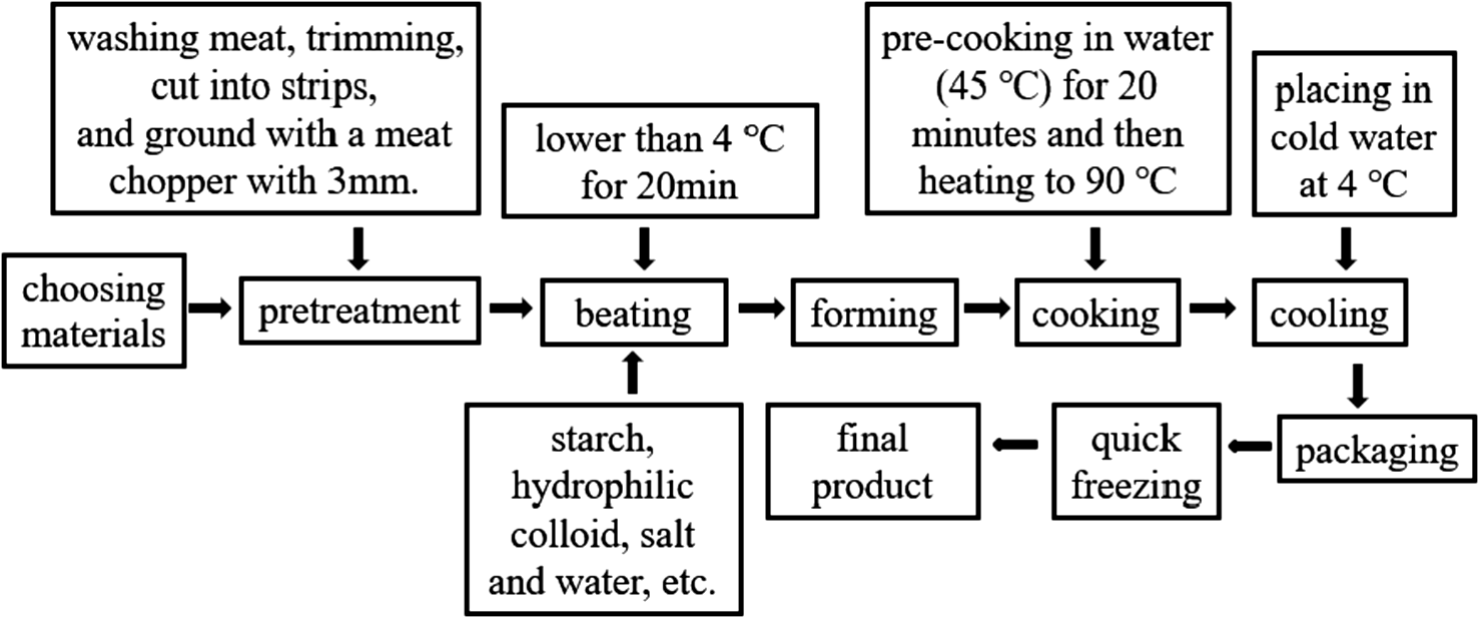
The technological process for beef meatballs’ preparation

The production process of BMs mainly includes mincing meat, beating, cooking, and preservation. These procedures are as follows: First, the beef and back fat were separately passed through a grinder, fitted with a 3‐mm diameter plate. Then, the minced meat was placed in a beating machine or a chopper. The seasonings containing salt, sugar, sodium tripolyphosphate, starch, ice water, back fat, and so on were put into the beating machine or the chopper and fully mixed. Additionally, the order of adding materials should be considered. In general, the raw meat is chopped and mixed first, and the ingredients such as salt and water are added. The purpose of this procedure is to maximize the extraction of salt‐soluble proteins, thereby forming emulsion and good texture of BMs. Third, the meat batters prepared are shaped into meatballs with diameters of 30 mm and cooked by different cooking methods. Finally, the appropriate preservatives, and packaging materials are utilized to effectively develop color stability and delay or restrain the oxidation process of BMs. Figure 2 shows the technological processes involved in BMs’ production. Table 1 shows the purpose of each ingredient and process.

**TABLE 1:** The purpose of each ingredient and process in beef meatballs

Ingredient and processing procedures	Purpose	Reference
Starch	Starch added to meat minced could improve the firmness of products, acting as a filler, while also improving gel strength	Wu et al. (2020)
Transglutaminase	Transglutaminase could reduce the amount of salt and phosphate usage, reduce or completely eliminate cooking losses, and create a gel structure in the foods in which it is present	Pietrasik and Chan (2002) Erdem et al. (2020)
Hydrocolloids	Hydrocolloids had been used in the meat products for film‐forming, fat‐replacing purposes, and stabilizing	Jimenez et al. (2013)
Nonmeat proteins	Nonmeat proteins were attributed to form the gel network structure of myofibrillar protein	Nishinari et al. (2014)
Salt	The role of NaCl in the solubilization of myofibrillar proteins is one of the main factors that make its addition mandatory in meat products. NaCl also reduces water activity, thus contributing to the preservation of the product	Choia et al. (2014)
Water	Water is attributed to the dissolution of ingredients	Nuray and Oz (2019)
Fat substitute	The fat substitute in meatballs improves the overall flavor based on the sensory evaluation	Ran et al. (2020)
Beating	Beating enlarges the contact surface between water and protein in muscle tissue, thereby increasing the retention ability of minced beef	Kang, Zou, et al., 2014
Cooking	Cooking improves the heat‐induced gelation ability of beef myofibrillar protein	Behannis et al. (2020)
Storage and preservation	Delay or restrain the oxidation process, effectively expending the shelf‐life of beef meatballs	Desvita et al. (2020)

## 3 EFFECTS OF RAW MATERIALS AND INGREDIENTS ON THE QUALITY OF BEEF MEATBALLS

### 3.1 Effect of distinct parts of beef on the quality of beef meatballs

The quality of beef is influenced by the postmortem aging time, varieties, and carcass parts. Besides, the part of beef is the main factor influencing beef quality. It is different from the content of components and nutrients in various parts, resulting in significant variations in the taste and flavor quality of meat products after processing (Zwolana et al., 2020). Also, there are differences in the water retention of muscle due to the differences in the beef parts. The gel properties (representing three‐dimensional (3D) network structures formed by the cross‐linking of protein molecules) of the muscle fiber proteins are affected by the muscle types. This property had been demonstrated to contribute to the water and fat retention capacity and the flavor adsorption efficacy of BMs. The main reason for the changes in gel attributes of various parts of meat is due to the different types of muscle fibers and the vitality of related endogenous enzymes. The gel retention and gel network structure of beef meatballs are positively correlated with the content of myofibrillar protein (MP). In recent years, some researchers have studied the suitability of processed meat products from different beef parts. Rhee et al. reported that different beef parts had various effects on the quality characteristics and suitable processing methods. The authors also noticed that the *biceps femoris* was more suitable for roasting than the *adductor*, *supraspinatus*, and *semitendinosus*. Besides, the biceps femoris had the lowest cooking loss rate and the best tenderness after processing, potentially caused by the shorter biceps femoris sarcomere and increasing protein degradation (Rhee et al., 2004). Existing studies have demonstrated that the sensory attributes (e.g., color, taste, odor, texture, and overall acceptance) of beef are significantly affected by the different parts of beef under the same processing conditions (Lawrence et al., 2001; Maria et al., 2012). In addition, the different parts of beef remarkably affected the flavors of beef, which could be explained by the fact that different parts of the meat had various volatile organic compounds (Legako et al., 2015; Xu et al., 2020). The mentioned research outcomes as well showed that the distinct parts of beef could significantly affect the quality of meat products. Conclusively, it is necessary to select the appropriate parts (round steak) of beef to prepare BMs with good quality. Also, attention should be paid to the combination of sensory and instrumental analysis (gas chromatograph‐time‐of‐flight mass spectrometer (GC‐ToF‐MS), electronic nose (E‐nose)), so as to provide the fundamental and supporting data for the industrialization and standardization of BM production.

### 3.2 Starch types

The BMs’ formula mainly includes lean meat, fat, water, starch, salt, spices, garlic, black pepper, sugar, monosodium glutamate, and so on. Many scholars have studied the effects of salt/fat substitutes, dietary fiber, and functional additives on flavor attributes, functionality, and yield of BMs (Kehlet et al., 2017; Liu et al., 2015; Petersson et al., 2014). Starch is one of the most widely used ingredients in meat‐based products, especially in BMs. The addition of starch can improve the firmness of meatballs, acting as a filler, while also enhancing the water‐holding capacity (WHC) and the gel strength. The starch with a high content of amylopectins, such as cassava, waxy corn, and potato starch, is generally selected and added to meatballs, due to its ability to improve the yield, stability, and water retention of meatballs. Furthermore, when meat products are heated, protein denaturation reduces the water‐binding capacity, while starch could absorb this part of water and produce gelatinization. On the other hand, water retention increased with increasing amylopectin content. Wu used potato, corn, cassava, and rice starch in myofibrillar protein–starch composite gels. Low‐field nuclear magnetic resonance (NMR) demonstrated that adding starch changed the relaxation area, but increased the relaxation time; indicating that the addition of starch could improve the water‐holding capacity (WHC) of the myofibrillar protein–starch composite gel, among which cassava starch was the best (Wu et al., 2020). Similarly, Serdaroglu et al. indicated that the incorporation of 2% or 4% corn starch (corn flour (CF)) resulted in an increment in protein content of cooked meatballs and had no detrimental effect on sensory properties, except appearance (Serdaroğlu & Değırmencioğlu, 2004). Wu et al. indicated that rice starch had a negative effect on the gel system, because the particles of rice starch were smaller and their distribution was not uniform, which could not support the entire protein network system as a filler (Wu et al., 2020). Compared with native starch, the functional properties of modified starch were significantly improved (Ryszard et al., 2021). Khalil noticed that patties formulated with the right amount of modified corn starch had higher cooking yield and moisture content (Khalil, 2000). It can be seen that different starch types have various effects on the quality of BMs. At present, there are almost no data on the application of modified starch in studying the gel properties of MP and the quality of BMs. Thus, the modified starch and other ingredients can be used in a particular proportion to study the effect of BMs.

### 3.3 Transglutaminase

The use of enzymes is an imperative method for improving the quality of meat products during processing. Protease is a useful additive of meat product, to modify the protein structure of food, tenderize the meat, and endow meat products with various excellent properties. Protease is divided into five categories. Transglutaminase (TGase) has the characteristics of water retention and strong gel ability. The enzyme can not only shorten the relaxation time of free water and enhance the binding capacity of meat emulsion on water molecules; but is also helpful to gel cross‐linking of minced meat and enhance cross‐linking between protein molecules, which may be linked to the formation of ε‐(‐glutamyl)‐lysine bond catalyzed by the TGase (Ahhmed et al., 2007). Erdem et al. indicated that the addition of TGase could significantly improve the springiness and chewiness of BMs (Erdem et al., 2020). Moreover, Tseng et al. observed that the yield of meatballs increased after supplementing TGase without any apparent effect on color. Texture, juiciness, and overall acceptability as analyzed by sensory evaluation were not statistically affected until the level of TGase reached 1.0% (Tseng et al., 2000). Notably, scanning electron microscopy (SEM) indicated firmer and more regular gel structures of meatballs with increasing TGase concentration, which is possibly due to the interaction between transglutaminase and myosin (Ahhmed et al., 2007; Erdem et al., 2020; Tseng et al., 2000). Therefore, the addition of TGase seems to be a beneficial method to enhance the quality and yield of BMs. Still the effect of enzymes on BMs depends on various conditions, such as enzyme reaction temperature, reaction time, and so on.

### 3.4 Hydrophilic colloid

Hydrophilic colloids are a promising class of natural agents that can be used to improve water retention and reduce protein loss in meat. Hydrophilic colloids are widely employed in meatballs under certain conditions, due to their functional properties such as thickening, gelling, and emulsifying abilities. For instance, scientific reports indicated that hydrophilic colloids such as flaxseed gum, carrageenan, sodium alginate, and gelatin have commonly been applied in meat products (Feng et al., 2018; Kang et al., 2020). As reported in the previous work of Ulu, the addition of carrageenan to low‐fat meatballs was more effective than guar gum for the textural properties after cooking (Ulu, 2006). Also, Zhang et al. showed that the addition of six kinds of powdered hydrophilic colloids (including chitosan, carrageenan, alginic acid, konjac glucomannan (KGM), sodium carboxymethylcellulose (CMC‐Na), and xanthan gum) at a lower dose could effectively inhibit the formation of 2‐amino‐1‐methyl‐6‐phenyl‐imidazole[4,5‐b]pyridine (PhIP) of the roast beef pie. Results in roast beef demonstrated that CMC‐Na was the most potent inhibitor against the formation of PhIP. In other words, the formation of heterocyclic amine in beef minced products could be inhibited by adding an appropriate amount of hydrophilic colloid (Zhang, Zhao, et al., 2020). So far, previous studies have demonstrated that the compound hydrocolloids has more obvious improvement in the nutritional, physical, and chemical quality of beef products compared with the single hydrophilic colloid (Renou et al., 2013). For example, Elizabeth et al. showed that the combination of locust bean gum, potato starch, and carrageenan could reduce the content of fat and sodium in minced beef products (Elizabeth & Alfonso, 2008). It can be seen that the compound hydrocolloids can significantly affect the quality of BMs. However, the effect of different compound hydrocolloids and their combination/interaction on the binding characteristics and quality of beef meatballs still need to be studied further. More extensive studies, also, on the mechanism of BMs should be conducted.

### 3.5 Nonmeat proteins

Nonmeat proteins, such as soybean separation protein, egg white protein, and whey protein, are incorporated into the processing of BMs. This is probably attributed to the nonmeat proteins that are effective on forming the gel network structure to bind water and fat, making BMs have high springiness and viscosity. The protein content in isolated soybean protein accounts for more than 90%. It effectively impacts on improving the water‐holding capacity (WHC), water/oil retention, yield, and gel strength of BMs (Nishinari et al., 2014). Odiase et al. showed that the addition of soy protein reduced cooking loss and enhanced water retention of BMs. Those authors also observed that meatballs with soy flour inclusions at 10% and 15% were most acceptable (Odiase et al., 2013). In addition, existing studies have suggested that the addition of soy protein and carrageenan can greatly enhance the gel strength and the water retention of beef minced (Gao et al., 2019; Hao et al., 2016). However, as a potential allergen, soybean protein may risk the health of the consumers. Moreover, the addition of a large amount of soybean protein can easily soften the textural characteristics and produce odor of the product. Furthermore, Serdaroğlu found that the addition of whey powder (WP) did not affect the sensory properties of meatballs. The addition of 2% or 4% WP significantly increased cooking yield and water‐holding capacity, regardless of the fat level (Serdaroğlu, 2006). The above studies also showed that the independent addition of different nonmeat proteins was beneficial to improve the quality of meat products. However, due to the differences in the structure and function of various nonmeat proteins, the effect of single use is often not ideal. The independent use of a single nonmeat protein may be detrimental to the quality of meatballs. This is mainly due to the differences and deficiencies in the structure and functional properties of various nonmeat protein types. Therefore, different types of nonmeat proteins to BMs were made up for the functional deficiency of each component. Besides, the directional changes in protein structure can be done through physical modification (e.g., microwave, pulsed electric field, ultrasonication) and chemical modification (such as macromolecular binding modification, chemical modification of protein side chain groups, and limited hydrolysis modification of peptide chain), which are beneficial to produce the satisfactory and acceptable BMs for the consumers.

### 3.6 Types of salt

The addition of salt can reduce the fat content and improve the yield, resulting in a unique flavor of BMs. The salt is conducive to the extraction of salt‐soluble proteins in muscles and promotes their fat and water‐binding ability. In common, the salt content is 2.0%–3.0% of BMs. Phosphate is an important quality improver for meat products. It can enhance the electrostatic repulsion between protein molecules. Besides, it can react with sodium chloride to enhance the solubility of myofibrillar protein and improve the water‐ and oil‐holding capacity of the minced meat system. However, a large amount of phosphate intake can affect human health. Therefore, the new processing techniques (such as ultrasonic treatment and high pressure treatment) combined with some phosphate alternatives (such as fiber, native starch, hydrophilic colloid, plant protein, and seaweed) can decrease the phosphate content in the meat products, which can inhibit the oxidation of the protein and unsaturated fatty acid to some extent. The water‐holding capacity (WHC), textural properties, and organoleptic characteristics of frankfurter sausages with 10% Ca‐ascorbate and 30% potassium lactate (K‐lactate) were close to the control (Choia et al., 2014). Besides, Cichoski et al. showed that the addition of 0.5% potassium chloride (KCl) to low phosphate meat emulsion was more stable at 25 kHz and 230 W by ultrasonic treatment for 27 min (Cichoski et al., 2019). However, more studies are needed to analyze the salty taste defect caused by the decrease of NaCl and evaluate the effect of this formula on the microbial quality of meat emulsion. Thus, the use of salt mixtures is an excellent method to reduce the NaCl content and maintain the quality of BMs.

### 3.7 Water addition

The addition of water in BMs is conducive to the dissolution of various raw materials so that they are easily mixed. In addition, starch can fully absorb the water and exert maximum gelatinization ability, thereby enhancing the gel ability of meatballs. Nevertheless, excess water may not be good for the formation of gels, causing the formation of weaker gels of minced meat. There are limited studies on the effect of water on the quality of BMs. Nuray indicated that heat treatment reduced the water content of BMs. The myofibril protein is contracted after heat treatment. Moreover, the volume between myofibril is reduced. This resulted in a decrease in the water‐holding capacity. Besides, shrinkage in the connective tissue around the muscle layer caused the compression of muscle fiber bundles at 56–62°C. Thus, it became easy to release free water from meat (Nuray & Oz, 2019).

### 3.8 Fat substitute

The fat in meatballs improves the taste, texture, and flavor and enhances its acceptability (Hu & Yu, 2015). High‐fat content in meatballs may increase the risk of cardiovascular disease. However, the reduction of fat content in meat products may have series of adverse effects on cooking loss rate and emulsion stability. Some researchers have reported on the effect of fat substitutes in meatballs. Öztürk et al. noted that 3% pumpkin seed kernel powder (PSK) could be recommended with minimal compositional and sensory changes as a fat replacer and functional ingredient in beef meatballs. The PSK flour reduced the fat content by about 9%–27% of BMs. Moreover, investigating the effects of fat replacers would be useful in providing the technological information and theoretical knowledge in developing low‐fat beef meatballs (Ran et al., 2020). Thus, improving fatty acid composition, reducing dietary fat content, and increasing dietary fiber content of meatballs, while enhancing the original meat product quality, have become the focus of research. Table 2 shows the advantages and disadvantages of common fat substitutes in BMs.

**TABLE 2:** Advantages and disadvantages of common fat substitutes in beef meatballs

Fat substitutes	Formulations of beef meatballs	Advantages	Disadvantages	Reference
Adzuki beans flour (ABF)	The total combined percentage of fat and corn flour from the meatball formulation was replaced with 50% (w/w) ABF	Replacement of fat with ABF especially at 50% (w/w) in the production of reduced fat meatballs resulted with acceptable sensory and better physicochemical properties compared to original meatballs	Their protein and carbohydrate contents remained the same compared to control	Aslinah et al. (2018)
Coconut flesh	Coconut flesh (10%)	Since both young Pandan and young Malayan Yellow Dwarf managed to reduce the fat content in the meatballs as compared to the control sample while maintaining the quality characteristics of the meatballs	There was no difference (*p* >.05) between all meatballs in terms of protein content	Khalid et al. (2021)
Pumpkin seed kernel powder (PSK)	Pumpkin seed kernel powder (3%)	The addition of PSK flour as well improved the fatty acid profile, reduced cooking loss, and improved polyunsaturated fatty acids’ (PUFAs’)/saturated fatty acids’ ratio of meatballs	PSK flour negatively affected redness and n−6/n−3 ratio of beef meatballs	Öztürk and Turhan (2020)
Gelatin and soluble dietary fibers (SDFs)	Gelatin and SDFs (20%)	Addition of the SDF–gelatin composite gels in the formulation increased the content of moisture, protein, Ca, Na, and ash in meatballs	Reduction of fat in meatballs has detrimental effects on meatball cooking characteristics	Niu et al. (2020)
Perilla seed	Perilla seed (10%)	The addition of 10% (w/w) Perilla seeds significantly improved the texture and content of polyunsaturated fatty acids (PUFAs), dietary fiber, and protein in meatballs	Adding an excessive amount led to the deterioration of hardness, elasticity, odor, and taste of meatballs	Ran et al. (2020)

## 4 EFFECT OF BEATING ON THE QUALITY OF BEEF MEATBALLS

Chopping and beating are two important treatment methods in the emulsification process, which are an important stage in manufacturing finely comminuted meat products. Beating machine simulates beating and blending meat with a stick, the traditional way the meatballs were produced in Asian communities. Kang et al. concluded that compared with chopping, the beating method resulted in higher L⁎‐values, changes in the *β*‐sheet content of the proteins and improved cooking yields at the same salt levels, which resulted in an improved product with a better texture (Kang, Wang, et al., 2014; Kang, Zou, et al., 2014). Kang et al. indicated that the beating process can produce low‐salt (1.0%) kung‐wan with the desirable sensory qualities of good hardness and elasticity (Kang, Zou, et al., 2014). The chopping method is used to produce frankfurters and other Western‐style meatballs. It can as well destroy the connective tissue membrane, break the peptide bonds of the protein molecules, and increase the polar groups, which can promote the ability of meat to absorb water. In addition, chopping can improve the internal structure of the meat emulsion, and afterward increase its uniformity and sliminess. The quality of beef minced is observably affected by the chopping time, speed, and temperature, and the order of addition of various ingredients. Boles et al. found that the color of meat products did not change when the chopping time was 5.7 min. This observation was possibly ascribed to the release of salt‐soluble protein in muscle tissue due to the suitable chopping time, which also enhanced the emulsion stability of meat batters (Boles et al., 1998). Barbut noticed that the hardness of the meat products increased with increasing chopping time (Barbut, 1989). However, too long time can induce the denaturation of protein and reduce the stability of the emulsion system, resulting in the alternations of the appearance in the product. At the same time, the chopping temperature also has a remarkable effect on the water and oil retention of the product. The optimum extraction temperature of myosin was 4–8°C. High temperature above 80°C can easily lead to the denaturation of myofibrillar protein (MP) and instability of the emulsion quality. Besides, the inability of soluble protein to completely encapsulate fat particles results in the failure of complete emulsification of fat. Thomas et al. assayed the effect of chopping temperature (16.3, 19.3, 27.4, and 34.8°C, respectively) on the product texture by the texture profile analysis (TPA). Analysis revealed an inverse relationship/correlation between the chopping temperature and shear force, chewiness and hardness. Results also showed an increase in cooking loss rate and thiobarbituric acid reactive substances (TBARS) value, whereas the reduction in moisture content was noticed with increasing chopping temperature (Thomas et al., 2007). Other studies have also shown that gel strength had the minimum value at 80°C, which may be due to prolonged heating time that negatively impacts the texture of MP gel (Huang et al., 2021). During the process of chopping, salt‐soluble protein is used as a continuous phase to fully wrap the dispersed phase, which promotes the springiness and chewiness of BMs. The mechanism of springiness and chewiness can be further explored in the follow‐up study, from the chopping and beating, and other factors. Only by grasping the key points of chopping and beating in practice can one produce high‐quality BMs. Table 3 shows the principles, advantages, and disadvantages of different beating methods.

**TABLE 3:** The principles, advantages, and disadvantages of different beating methods

Methods	Advantages	Disadvantages	Reference
Beating	The beating process formed more continuous and compact structures at the same salt content compared with chopping. Beating process allowed for the production of low‐salt and higher quality kung‐wans	When the beater beating machine speed is too fast, the meat is rapidly increased, and the amount of dissolution of myoblast protein is reduced, which is not conducive to the formation of the gel structure	Kang, Wang, et al. (2014)
Chopping	Meatballs produced by the vacuum bowl cutter have better softness and tenderness	Chopping process resulted in worse texture than beating at the same salt level	Kang, Zou, et al., 2014

## 5 EFFECT OF COOKING METHODS ON THE QUALITY OF BEEF MEATBALLS

### 5.1 Traditional cooking methods

The cooking methods used in meatball processing mainly include boiling, oven cooking, frying, and a new processing approach. In general, the BMs are precooked in water (45°C) for 20 min and then heated to 80°C. In this process, meatballs are gelatinized at low temperature and then cooked slowly at high temperature. However, myosin formed a complex and weak network structure when BMs were directly exposed to high temperature. Besides, the BMs should be fetched on time and placed in cold water at 10°C after heat treatment. The BMs could be cooled to reabsorb the water lost during the cooking process, which could prevent the surface of the meatballs from wrinkling and browning. Different cooking methods may lead to different effects. Table 4 shows the advantages and disadvantages of BMs cooked by different traditional cooking methods.

**TABLE 4:** The principles, advantages, and disadvantages of different traditional cooking methods

Cooking	Advantages	Disadvantages	Reference
Boiling	This may be results in the sufficient time for the reaction between protein molecules at lower temperature. The elasticity of beef meatballs gradually increases with the increase of temperature	Texture profile analysis (TPA) hardness of meatballs with fiber, cooked in boiling water, was lower compared to oven‐baked and pan‐fried	Behannis et al. (2020)
Oven	The highest cooking yield was found in samples cooked in the oven. Loss of nutrients after cooking was lower for oven‐baked compared to boiling	It took more time to achieve the same level of inactivation in the oven compared with the fryer	Mena et al. (2020) Porto et al. (2016)
Frying	Compared with the deep‐fat frying group, hot air‐fried giant salamander meatballs had higher L∗, elasticity, and hardness	Compared with the deep‐fat frying group, hot air‐fried giant salamander meatballs had lower fat content, b∗value, a∗, and yield	Mena et al. (2020)

### 5.2 Novel cooking methods

The new cooking methods include ohmic heating, ultrasonic‐assisted (US) processing, and infrared final cooking, moderate electric field (MEF), and so on. Ultrasonication is a useful tool for preparing meat products (José et al., 2019). US can also improve the stability and technological quality of meat emulsions. Besides, it promotes the dissolution and penetration of flavor substances into raw materials, thereby improving the flavor of meat products. In addition, the application of ultrasound‐aided processing is conducive for improving the tenderness and water retention of meatballs. Wang et al. also indicated that ultrasonic‐assisted frying to cook meatballs had a higher water‐holding capacity (WHC) than traditional frying. This was possibly credited to the network of fried meatballs that presented a regular porosity state, thus inhibiting water evaporation. This may also be linked to the mechanical effect of ultrasonication, transforming more free water into immovable water. Additionally, the ultrasonic treatment group at 80°C shortened the cooking time by 1–3 min compared with the traditional frying group (Wang et al., 2019). Ultrasonic‐aided technology has an excellent application prospect as an efficient and energy‐saving new cooking method. This technology can reduce the use of food additives such as carrageenan and soybean protein without affecting the quality of minced products. However, ultrasonication should be noticed during the treatment because the ultrasonic cavitation produces the air bubbles. The disappearance of these bubbles can release large amounts of energy and lead to the increase of temperature. Thus, it is necessary to control the meat temperature and the state of the myofibrillar protein during meat processing (Rohman et al., 2011). Ohmic heating, a well‐known electroheating technique, is used in commercial‐scale operations for processing a number of food products. Higher yields, shorter processing times, maintenance of the color, and nutritional value of foods are some of the advantages of ohmic cooking when compared to conventional heating (Filiz & Coskan, 2004). Sengun et al. found that ohmic heating could eliminate *Salmonella* and *Staphylococcus aureus* in precooked meatballs, while it could not effectively eliminate *Listeria monocytogenes* cells (Sengun et al., 2014). Infrared cooking is of interest to the food ingredients and the processed meat sector. Infrared radiation has intrinsic advantages such as keeping oven temperatures and humidity at low values. A further advantage of this method is the ease with which heat can be applied evenly over a broad surface area (Sheridan & Shiton, 1999). Kendirci indicated that precooking followed by infrared cooking may be regarded as a safe cooking method of meatballs from a polycyclic aromatic hydrocarbon (PAH) contamination point of view (Perihan et al. (2014). In addition, the use of moderate electric field (MEF) heating as an emerging cooking inactivation technology has gained considerable attention in more recent times. Therefore, it is necessary to consider the combination of cooking methods in further research (Sengun et al., 2014). Table 5 shows the principles, advantages, and disadvantages of different novel cooking techniques. Further studies should be carried out that optimize parameters for meatball cooking while putting into practice ohmic and infrared heating combination system. In addition, it is advisable to set the optimum cooking parameters and the improvement of the quality of meatballs into practice when meatballs were prepared.

**TABLE 5:** The principles, advantages, and disadvantages of different novel cooking techniques

Cooking	Advantages	Disadvantages	Reference
Ohmic cooking	In ohmic cooking, heat generation was homogeneous, a uniform temperature increase within the meat sample could be obtained. Ohmic cooking seems to be the best cooking method in terms of retaining fatty acid in meatballs enriched with flaxseed flour	The distinctive crust layer was not formed at the meatball surface during ohmic cooking, so physical retention of fat and water was difficult	Turp (2016)
Ultrasonic‐assisted frying	The application of ultrasonic‐assisted frying could accelerate the speed of free fatty acids’ (FFAs) oxidation and would further promote the generation of volatile flavor compounds and improve the flavor quality of meatballs	Longer ultrasound (40 min) treatment resulted in a decrease in hardness, G′ value, and water‐holding capacity (WHC) of meat batter (MB)	Zhang, Zhao, et al. (2020); Li et al. (2015)
Infrared final cooking	Infrared cooking, which was mainly effective for surface heating, could be applied as a final cooking method to improve the quality characteristics of ohmically precooked beef meatballs	The cooking loss rate of the meatballs increased at the extended application duration of infrared heating	Turp et al. (2016); Perihan et al. (2014)
Moderate electric field	Moderate electric field could be used to cook meat in a shorter time and with a reasonably low energy input while producing a product which was comparable in quality to conventionally cooked meatballs	Further work was required to investigate the effect of moderate electric field cooking methods on consumer acceptance, sensorial properties, and the availability of nutrients in the food products under investigation	Bedane et al. (2021)

## 6 EFFECT OF STORAGE AND PRESERVATION PROCESS ON THE QUALITY OF BEEF MEATBALLS

### 6.1 Antistaling agent

The addition of antistaling agent is one of the acceptable methods used to delay and/or prevent the oxidative deterioration in processed meat products. Extracting nisin lactate, sodium lactate, butylated hydroxytoluene, butylated hydroxyanisole, and extraction of the plant materials (including herbs, perfumes, fruits, and vegetables) are widely used in meatballs to improve the quality and prolong the shelf‐life (FahadY et al., 2020; Khaoula et al., 2020; Oz, 2019; Prommachart et al., 2020). Different types of preservatives have other antibacterial mechanisms. Besides, BMs are susceptible to bacterial contamination, which makes it conducive to the rapid development of stale or rancid flavor during storage and preservation. Recently, many researchers have focused on applying natural active substances to the antioxidant activity of beef meatballs. Table 6 shows the processing methods and the results of common preservatives used in BM production.

**TABLE 6:** Common preservative active ingredients in beef meatballs

Active ingredients	Formulations of beef meatballs	Processing method	Storage condition	Result	Reference
Pomegranate peel nanoparticles	Pomegranate peel nanoparticles (1% and 1.5%)	Meatballs were formed by hand, sealed with one layer of a wrapping film	Stored at 4 ± 1°C for 15 days	Peroxide value↓, thiobarbituric acid (TBA) reactant value↓, total volatile basic nitrogen content↓, phenolic content↑, antioxidant and antimicrobial properties↑, lipid and protein oxidation↓, water‐holding capacity (WHC) ↑, cooking yield↑	Morsya et al. (2018)
Pomegranate seed extract	Pomegranate seed extract (0.5%)	Baking in the oven, pan frying, charcoal baking, and frying	Stored at −18°C, prior to analysis, meatball samples were thawed in a refrigerator at 4°C for 12–24 hr	Total heterocyclic aromatic amine formation was reduced by 46% and 39% in beef meatballs cooked by deep‐fat frying and charcoal‐barbecue, respectively	Keşkekoğlu and Ürenb (2014)
*Moringa oleifera* leaf extract	*Moringa oleifera* leaf extract (0.1%, 0.2%, and 0.3%)	When internal temperature of beef meatballs reached 71°C, then cooking was finished	Stored at −20°C for 60 days	Cooking loss↓, free fatty acid (FFA) value↓, peroxide value (POV)↓, thiobarbituric acid reactive substance (TBARS) value↓, microbial values↓, total coliform count (TCC) value↓, the total yeast–mold count↓, juiciness↑, overall acceptability↑	Islam et al. (2018)
Nutmeg extract	Nutmeg extract (0.2%) and nutmeg powder (0.2%)	Boiling (at 100°C for 22 min), pan‐roasting (at 180°C for 5 min), convection oven (at 120°C for 20 min), and microwave oven (at 2450 MHz for 70 s)	Stored at −18°C frozen storage for 1 and 2 months separately	Overall acceptability↑, color and texture values↑, oxidative (lipid and protein) stability↑, reheating loss↓, hardness value of texture↓, rancid off‐flavor↓	Rashida et al. (2020)
Pomegranate peel extract	Pomegranate peel extract (1%) and freeze‐dried powder (0.5%)	Beef meatballs were shaped by hand and packed in oxygen‐permeable bags	Stored in the dark at −18 ± 1°C for 6 months	Lipid and protein oxidation↓, rancid smell↓, sensory properties↑, malondialdehyde↓, peroxide↓, carbonyl formation↓, loss of total protein solubility↓, sulfhydryl groups↓	Turgut et al. (2017)
Ginger	Garlic, onion, red chilli, paprika, ginger, and black pepper powder (0.5%)	Frying at 180°C for 3 min until the core temperature of 71.8–73.1°C was reached	Cooked meatballs were chilled at 4°C overnight, followed by storage at −18°C	All the spices powder reduced the formation of total heterocyclic amines (HCAs), while ginger powder achieved the highest inhibition efficiency compared with all other spices	Lu et al. (2018)
Black pepper	Spreading on the surface of meats for 12 hr prior to frying as 1% (w/w)	Frying at 175°C, 200°C, or 225°C for 7.5 min	Stored at −18°C until analyzed, they were thawed at 4°C for 12–24 hr prior to use	Thiobarbituric acid reactive substance (TBARS) value↓, cooking loss↓, heterocyclic aromatic amines↓	Oz and Kaya (2009)

Note

↑, increased after preservative active ingredients’ addition; ↓, decreased after preservative active ingredients’ addition; →, unchanged after preservative active ingredients’ addition.

These results indicated that the addition of preservatives in the processing of meatballs could effectively inhibit the oxidative deterioration of beef meatballs, and prolong the shelf‐life of products without affecting the sensory characteristics. Future research may consider the combination of a variety of natural preservatives. Besides, the application of natural antioxidant instead of synthetic antioxidants can be considered at both industrial and small‐scale levels to enhance the quality of BMs.

### 6.2 Packing material

Packaging meatballs can reduce the incidence of foodborne diseases and significantly extend the storage period of meatballs. In addition, the packaging system can affect MP associated with beef tenderness. Edible packaging is generally used to improve the mechanical properties of the food. Edible composite packaging materials can be applied to meatball packaging because of its green pollution‐free and good preservation effect (Desvita et al., 2020). Many different substances have suitable properties for use as a coating or film. Table 7 shows the results of meatballs treated with different packaging materials.

**TABLE 7:** Processing meatballs with different packaging materials

Packaging materials	Processing mode	Results	Reference
Edible chitosan	(1) Buy commercial shrimp shell chitosan, with a minimum deacetylation degree of 90% (2) Twenty grams of chitosan was mixed with 1000 ml of distilled water and stirred for 10 min at 60°C (3) Ten milliliters of glacial acetic acid was added to the mixture, which was stirred for 1 h	Lipid oxidation↓, *Bacillus cereus*↓, *Staphylococcus aureus*↓, *Escherichia coli*↓, *Pseudomonas fluorescens*↓	Sweetie et al. (2013)
Edible film from bovine split hide gelatin	(1) Gelatin 0, 5, and 10% (b/v) (2) Making suspension in distilled water (3) Heating and stirring (55°C, 30 min) (4) Addition of glycerol (50% w/w) 5) Heating at 70°C for 15 min 6) Edible coating solution:	pH value→, soluble protein→, water‐holding capacity (WHC)→, microorganisms↓	Wulandari et al. (2016)
Whey protein edible films	(1) Whey protein isolate (5% wt/vol) was dissolved in distilled water, and glycerol (5% wt/vol) was added (2) Solutions were heated to 90 ± 2°C while being stirred continuously for 30 min and cooling to room temperature (3) The film solutions were filtered through a layer of cheesecloth. (4) Added phytochemicals from *Laurus nobilis* L. and *Salvia officinalis* extracts into film solutions	Thiobarbituric acid (TBA) values↓, para‐anisidine value↓, total phenolic compound content↑, peroxide value↓, conjugated dienes’ value↓, lipid oxidation↓, lipid hydrolysis↓	Akcan et al. (2017)

Note

↑, increased after packaging materials’ addition; ↓, decreased after packaging materials’ addition; →, unchanged after packaging materials’ addition.

Thus, it is a feasible method to select some useful packaging materials in the production of meatballs. Packaging meatballs effectively inhibit the growth of microorganisms and the oxidative deterioration of meatballs, without affecting the sensory characteristics of meatballs.

### 6.3 Packaging method

At present, many researchers have studied the effect of different packaging methods on meatball quality (Li et al., 2020). Yilmaz and co‐workers observed that modified atmosphere packaging (MAP) could improve the quality properties and inhibit microbial growth of meatball during storage (Ozturk et al., 2010; Yilmaz & Demirci, 2010). Meatball samples can be stored without any microbiological problem for 7 days at 4°C at MAP (containing 65% N_2_, 35% CO_2_) (Yilmaz & Demirci, 2010). However, Karpińska‐Tymoszczyk studied the effect of vacuum and modified atmosphere packaging on the quality of turkey meatballs stored at 4°C. The authors noted that the vacuum packaging prevented the oxidation of meatballs more effectively than MAP (Karpińska‐Tymoszczyk, 2013). Scientific reports on the lipid oxidation of meat and meat products by MAP are relatively common, while studies on protein oxidation are limited. It can be concluded that packaging materials and methods play a key role in extending the shelf‐life of beef meatballs. The packaging approaches are more promising or beneficial to BMs. Thus, different packaging and preservation methods can be applied in BMs’ product to extend the shelf‐life and ensure the quality and flavor of meat products.

In addition, sensory characteristics of meat products are very important in the acceptance and selection by consumers. At present, some meat products have established a set of sensory evaluation systems (Sasaki et al., 2014). For example, Saldaña et al. performed a descriptive analysis of bacons smoked with woods from liquid and reforestation smokes. This result showed that the sensory profile of the traditional bacon was affected by the smoking process (Saldañaa et al., 2018). However, it is necessary to establish the detailed sensory evaluation system and the appropriate standard protocols in obtaining quality BMs. Therefore, quantitative descriptive analysis by a trained sensory panel is used to examine the sensory characteristics of BMs. The sensory evaluation system of BMs is established by multivariate statistical analysis. In addition, the flavor compounds and texture of BMs are analyzed. The purpose is to establish the correlation between aroma attributes and flavor of BMs, and the correlation between texture attributes and texture of BMs. It may be used by food companies and other stakeholders to understand the changes in sensory characteristics of BMs due to ingredients, cooking methods, and storage and preservation process.

## 7 CONCLUSION AND FORESIGHT

With the increasing demand of convenience, and nutritious foods, beef products have become a popular food in the meat industry. This demand initiates the development of BMs in industry. In this paper, the effects of different factors which include ingredients, beating, cooking methods, and storage and preservation on the quality of BMs were comprehensively explained. Various factors on sensory, physical, chemical, nutritional, and safety attributes of beef meatballs were discussed. Beating, salt, starch, the order of ingredients, and cooking play a significant role in gel formation of MP, and emulsification stability of BMs with a unique taste profile, and better water–fat binding. This is vital to assure the quality of BMs. However, some BMs on the market showed low quality, which limited the rapid development of BM in meat industries. Therefore, in a follow‐up study, BMs from the following aspects will be/need to be considered: (1) modification of MP by nonthermal processing technology to improve the gel properties of beef; (2) detecting adulteration in beef meatballs, and doing market research to look for deficiencies in the BMs industry; (3) assay sensory analysis of BMs, and establish the suitable sensory evaluation system of BMs by consumer preference experiments; (4) explore the beating mechanism during the process of meatballs and excavate the fast and efficient processing methods of BMs; (5) study the combined effect of novel techniques (ultrasonication +ohmic heating) during the cooking process on the yield and quality of BMs. Further research will also be done to investigate the combined application of different packaging methods and antistaling agent in BMs. Finally, this review may create new opportunities for providing guidance for the industrial production and quality assurance of beef meatballs.

## CONFLICT OF INTEREST

We declare that there are no known competing financial interests or personal relationships that could have appeared to influence the work reported in this paper.

## Data Availability

Data sharing is not applicable to this article as no datasets were generated or analyzed during the current study.
